# Gene Silencing in Crustaceans: From Basic Research to Biotechnologies

**DOI:** 10.3390/genes4040620

**Published:** 2013-11-07

**Authors:** Amir Sagi, Rivka Manor, Tomer Ventura

**Affiliations:** 1 Department of Life Sciences and the National Institute for Biotechnology in the Negev, Ben-Gurion University, P.O. Box 653, Beer Sheva 84105, Israel; E-Mail: mnor@bgu.ac.il; 2 Faculty of Science, Health, Education and Engineering, GeneCology Research Centre, University of the Sunshine Coast, 4 Locked Bag, Maroochydore, Queensland 4558, Australia; E-Mail: tventura@usc.edu.au

**Keywords:** crustaceans, gene function, RNA interference, humoral defense mechanisms, androgenic gland, insulin-like peptide, biotechnology

## Abstract

Gene silencing through RNA interference (RNAi) is gaining momentum for crustaceans, both in basic research and for commercial development. RNAi has proven instrumental in a growing number of crustacean species, revealing the functionality of novel crustacean genes essential among others to development, growth, metabolism and reproduction. Extensive studies have also been done on silencing of viral transcripts in crustaceans, contributing to the understanding of the defense mechanisms of crustaceans and strategies employed by viruses to overcome these. The first practical use of gene silencing in aquaculture industry has been recently achieved, through manipulation of a crustacean insulin-like androgenic gland hormone. This review summarizes the advancements in the use of RNAi in crustaceans, and assesses the advantages of this method, as well as the current hurdles that hinder its large-scale practice.

## 1. Gene Silencing is Gaining Momentum in Crustaceans

In 1998, Fire and colleagues [1] were the first to reveal the induction of RNA interference (RNAi) by exogenous application of double-stranded RNA (dsRNA) in a metazoan species. This work has since revolutionized the field of molecular genetics, prompting a plethora of studies applying dsRNA for gene knock-down studies through RNAi in a variety of metazoan species [2], leading to the awarding of Andrew Z. Fire and Craig C. Mello with the Nobel Prize in Physiology or Medicine in 2006 [3]. RNAi is an endogenous mechanism for highly specific post transcriptional silencing of gene expression. The active components, small interfering RNAs (siRNAs) are generated endogenously to moderate gene activity, but can be generated by exogenous administration of siRNAs, short hairpin-loop RNAs (shRNAs) or long dsRNA that is cleaved into siRNAs by an enzyme called Dicer. These siRNAs then serve as a sequence specific locator of the target RNA as part of the endonuclease activity of the RNA-induced silencing complex (RISC) [4,5,6]. Up until 2005, only a handful of pioneering reports had evaluated the use of RNAi in crustaceans [7,8,9,10,11]. These preceded a few dozen other published studies within the following eight years, most reporting the exogenous application of dsRNA. It is thus evident that RNAi-based research in crustaceans is gaining momentum, and is becoming an integral scientific component for rapid and reliable study of crustacean gene functions. RNAi enables transient knock-down of specific gene expression, without the need to genetically modify the studied organisms. In comparison to *in vivo* studies applying RNAi in model organisms [12], this field is still in its infancy in crustaceans, perhaps owing to the fact that the administration methods (lack of oral administration, referred to in Section 3.3) have not reached a stage where large-scale studies are optional. As next generation sequencing technologies become more affordable and more commonly used, it is estimated that crustaceans will not lag far behind model organisms, for which genomic data availability simplifies tailoring of siRNAs and multi-gene research approach is feasible.

*Crustacea* is a large (~67,000 known species) and diverse Subphylum of arthropods [13], populating various niches ranging from terrestrial to marine, from flourishing tidal zones to extremely harsh and low populated environments such as thermal vents. Due to their extremely wide dispersal they serve as model organisms to monitor toxicity [14] and environmental changes [15], while on the other hand, their hardiness and adaptability place them among the worst known invasive species [16,17]. Crustaceans are considered ancestral to insects [18,19] and are thus an important group for arthropod evolution studies. Many crustacean species, primarily from the order *Decapoda*, are commercially important for human consumption [16], while the entire aquaculture industry heavily relies on stable, continuous supply of but a few branchiopod crustacean (*i.e.*, *Artemia*) species [20]. 

Most RNAi studies in crustaceans performed to date were on commercially important decapod species, and will be elaborately discussed in this review. However, the employment of crustacean model organisms—such as the branchiopod *Daphnia pulex*, whose genome was fully sequenced and annotated [21]—might change this trend.

In crustaceans, dsRNA injections have proven to be the most efficient method for RNAi induction. Although this injection-based methodology was found to be successful in the laboratory, in order to enable rapid, large-scale RNAi experiments, other delivery techniques must be devised. One such large-scale administration method of RNAi is used in the plathyhelminth *Caenorhabditis elegans*, where feeding with dsRNA-producing bacteria proved to be effective [22]. Unlike the time-consuming and laborious injections that are sometimes confined by the size of the organism, the option of RNAi administration via feeding could potentially be applied for manipulating a wide array of genes in many individuals at the same time. Attempts to devise a more efficient administration method were made primarily in the context of anti-viral defense in crustaceans and this is thus discussed as part of the section dedicated to this subject (Section 3.2 and Section 3.3). 

Solving the delivery hurdle might be achieved by suggested methods described for other organisms [23]. A case study of RNAi biotechnology application in aquaculture is given in Section 4, delineating how prawn monosex populations were generated for the first time through RNAi-based biotechnology at a large scale aquaculture industry. Other plausible applications for RNAi in aquaculture of crustaceans might be for the control and synchronization of the molt cycle, eradication of invasive species and immune defense.

## 2. Understanding the Functionality of Novel Crustacean Genes through Gene Silencing

Gene silencing has contributed to our understanding of the functionality of novel crustacean genes with importance to development, growth, metabolism and reproduction, including genes encoding structural exoskeletal proteins, eyestalk neuropeptides and receptors. This section reviews studies that have used RNAi for this purpose (not including viral-related genes, which will be discussed in Section 3). The list of functional genomic cases reviewed in this section is summarized in Table 1.

### 2.1. Genes Related to Biomineralization

Biomineralization in crustaceans plays a key role in calcium metabolism during molting: the periodic shedding and replacement of the exoskeleton. Several crustacean species form temporary extracellular calcium carbonate storage deposits during the premolt stage [24]. In freshwater crayfish, including the Australian red-claw crayfish *Cherax* *quadricarinatus*, these deposits are in the form of a pair of disc-like structures, known as gastroliths, which are located on either side of the stomach wall [25,26]. Recently, the role of two gastrolith proteins (GAPs) in *C.* *quadricarinatus*, GAP 65 and GAP 10, in the development of gastroliths was demonstrated using dsRNA injections. GAP 65, which was extracted from late premolt gastroliths of *C.* *quadricarinatus*, is a calcium-binding glycoprotein, predicted to bind chitin. In a functional assay of GAP 65, this protein was suggested to play a dual role. Using dsRNA to silence the gene encoding *GAP 65* *in vivo* demonstrated its involvement in both the construction of extracellular matrix and deposition of calcium. In this study irregularities in gastrolith morphologies were manifested following the knockdown of *GAP 65* [27]. Silencing of *GAP 10* *in vivo* resulted in a prolonged premolt stage and in the development of gastroliths with irregularly rough surfaces. These findings suggest that *GAP 10* may be involved in the assembly of the gastrolith chitin-protein-mineral complex, particularly in the deposition of amorphous calcium carbonate [28]. These two studies demonstrate how within a relatively short period, functionality of newly discovered genes can be assessed using RNAi, unveiling different aspects in the assembly of a previously over-looked organ.

**Table 1:** Study of novel crustacean genes using RNA interference (RNAi).

Field of Research	RNAi administration method	Model Organinsm	Order	Year	Authors	Title
Biominerology	dsRNA injection	*Cherax quadricarinatus*	*Decapoda*	2008	Shechter *et al.*	A gastrolith protein serving a dual role in the formation of an amorphous mineral containing extracellular matrix
Biominerology	dsRNA injection	*Cherax quadricarinatus*	*Decapoda*	2010	Glazer *et al.*	A protein involved in the assembly of an extracellular calcium storage matrix
Development/differentiation	dsRNA injection	*Artemia franciscana*	*Anostraca*	2006	Copf *et al.*	Knockdown of spalt function by RNAi causes de-repression of Hox genes and homeotic transformations in the crustacean *Artemia franciscana*
Development/differentiation	Chemicaly modified siRNA oligo injection	*Parhyale Ultrabithorax*	*Amphipoda*	2009	Liubicich *et al.*	Knockdown of *Parhyale Ultrabithorax* recapitulates evolutionary changes in crustacean appendage morphology
Development/differentiation	dsRNA injection	*Daphnia magna*	*Cladocera*	2011	Kato *et al.*	Development of an RNA interference method in the cladoceran crustacean *Daphnia magna*
Development/differentiation	dsRNA injection	*Litopenaeus vannamei*	*Decapoda*	2008	Hui *et al.*	Characterization of the putative farnesoic acid *O*-methyltransferase (LvFAMeT) cDNA from white shrimp, *Litopenaeus vannamei*: Evidence for its role in molting
Metabolism	dsRNA injection	*Litopenaeus vannamei*	*Decapoda*	2010	Soñanez-Organis *et al.*	Silencing of the hypoxia inducible factor 1 -HIF-1- obliterates the effects of hypoxia on glucose and lactate concentrations in a tissue-specific manner in the shrimp *Litopenaeus vannamei*
Growth	dsRNA injection	*Penaeus monodon*	*Decapoda*	2011	De Santis *et al.*	Growing backwards: an inverted role for the shrimp ortholog of vertebrate myostatin and GDF11
Metabolism	dsRNA injection	*Procambarus clarkia*	*Decapoda*	2011	White *et al.*	Characterization of sarcoplasmic calcium binding protein (SCP) variants from freshwater crayfish Procambarus clarkii
Metabolism	dsRNA injection	*Litopenaeus schmitti*	*Decapoda*	2006	Lugo *et al.*	Molecular cloning and characterization of the crustacean hyperglycemic hormone cDNA from *Litopenaeus schmitti*
Reproduction	dsRNA injection	*Metapenaeus ensis*	*Decapoda*	2007	Tiu and Chan	The use of recombinant protein and RNA interference approaches to study the reproductive functions of a gonad-stimulating hormone from the shrimp *Metapenaeus ensis*
Molt	dsRNA injection	*Cherax quadricarinatus*	*Decapoda*	2012	Pamuru *et al.*	Stimulation of molt by RNA interference of the molt-inhibiting hormone in the crayfish *Cherax quadricarinatus*
Osmo-regulation	dsRNA injection	*Litopenaeus vannamei*	*Decapoda*	2007	Tiu *et al.*	The LvCHH-ITP gene of the shrimp (*Litopenaeus vannamei*) produces a widely expressed putative ion transport peptide (LvITP) for osmo-regulation
Reproduction	dsRNA in tissue culture	*Penaeus monodon*	*Decapoda*	2008	Treerattrakool *et al.*	Molecular characterization of gonad-inhibiting hormone of *Penaeus monodon* and elucidation of its inhibitory role in vitellogenin expression by RNA interference
Reproduction	dsRNA injection	*Penaeus monodon*	*Decapoda*	2011	Treerattrakool *et al.*	Induction of Ovarian Maturation and Spawning in *Penaeus monodon* Broodstock by Double-Stranded RNA
Reproduction	Feeding with dsRNA enriched Artemia	*Penaeus* *monodon*	*Decapoda*	2013	Treerattrakool *et al.*	Silencing of gonad-inhibiting hormone gene expression in *Penaeus monodon* by feeding with GIH dsRNA enriched Artemia
Reproduction	dsRNA injection	*Penaeus* *monodon*	*Decapoda*	2011	Sathapondecha *et al.*	Potential roles of transglutaminase and thioredoxin in the release of gonad-stimulating factor in *Penaeus monodon*: Implication from differential expression in the brain during ovarian maturation cycle
Growth	dsRNA injection	*Macrobrachium rosenbergii*	*Decapoda*	2013	Sharabi *et al.*	Dual function of a putative epidermal growth factor receptor in the decapod crustacean *Macrobrachium rosenbergii*
Reproduction	dsRNA injection	*Penaeus monodon*	*Decapoda*	2008	Tiu *et al.*	From hepatopancreas to ovary: molecular characterization of a shrimp vitellogenin receptor involved in the processing of vitellogenin
Molt	dsRNA injection	*Fenneropenaeus chinensis*	*Decapoda*	2009	Priya *et al.*	Molecular characterization and effect of RNA interference of retinoid X receptor (RXR) on E75 and chitinase gene expression in Chinese shrimp *Fenneropenaeuschinensis*
Molt	dsRNA injection	*Fenneropenaeus chinensis*	*Decapoda*	2010	Priya *et al.*	Molecular characterization of an ecdysone inducible gene E75 of Chinese shrimp *Fenneropenaeus chinensis* and elucidation of its role in molting by RNA interference
Organ regeneration	dsRNA injection	*Uca pugilator*	*Decapoda*	2013	Das and Durica	Ecdysteroid receptor signaling disruption obstructs blastemal cell proliferation during limb regeneration in the fiddler crab, *Uca pugilator*
Reproduction	dsRNA injection	*Carcinus* *maenas*	*Decapoda*	2011	Nagaraju *et al.*	Molecular cloning and sequence of retinoid X receptor in the green crab *Carcinus maenas*: a possible role in female reproduction
Sexual differentiation	dsRNA injection	*Daphnia magna*	*Cladocera*	2011	Kato *et al.*	Environmental sex determination in the branchiopod crustacean *Daphnia magna*: deep conservation of a Doublesex gene in the sex-determining pathway
Sexual differentiation	dsRNA injection	*Cherax quadricarinatus*	*Decapoda*	2012	Rosen *et al.*	A sexual shift induced by silencing of a single insulin-like gene in crayfish: ovarian upregulation and testicular degeneration
Sexual differentiation	dsRNA injection	*Macrobrachium rosenbergii*	*Decapoda*	2009	Ventura *et al.*	Temporal silencing of an androgenic gland-specific insulin-like gene affecting phenotypic gender differences and spermatogenesis
Sexual differentiation	dsRNA injection	*Macrobrachium rosenbergii*	*Decapoda*	2012	Ventura *et al.*	Timing sexual differentiation full functional sex seversal achieved through silencing of a single insulin like gene in the prawn *Macrobrachium rosenbergii*
RNAi	dsRNA injection	*Penaeus monodon*	*Decapoda*	2008	Dechklar *et al.*	Characterization of Argonaute cDNA from *Penaeus monodon* and implication of its role in RNA interference
RNAi	dsRNA injection	*Marsupenaeus japonicus*	*Decapoda*	2012	Wang *et al.*	TRBP and eIF6 Homologue in *Marsupenaeus japonicus* Play Crucial Roles in Antiviral Response

### 2.2. Genes Related to Development, Differentiation and Metabolism

Hox genes specify segmental identities and organ patterning in arthropods by forming a gradient of expression along body axes during embryo development [29]. Spalt genes are associated with Hox gene function, with varied association in diverse species, acting as cofactors, regulators or targets of different Hox paralogues. Copf *et al.* [30] studied the role of Spalt in the branchiopod crustacean *Artemia franciscana*. Using RNAi, they showed that knocking down *Spalt* expression results in various segmental anomalies. Based on the silencing phenotypes, combined with the spatial-temporal expression pattern of *Spalt* and prior knowledge of Hox genes, the authors suggested that *Spalt* acts as a repressor of Hox gene expression in *A. franciscana* where unleashing the expression of Hox genes in an uncontrolled pattern, through *Spalt* silencing, generates stochastic aberrations of segmentation. 

In another study, the Hox gene *Ultrabithorax* (*Ubx*, [31]) was shown to regulate the number of maxillipeds in the amphipod *Parhyale hawaiensis*. Reduction of *Ubx* expression via siRNA injections, led to a phenotype of additional maxillipeds, which persisted in adults, supporting the key role of Ubx in defining crustacean appendage boundaries. Since the new morphological pattern was similar to that seen in other crustacean species, this alteration was interpreted as an insight into the mechanism of morphological evolution [31]. One plausible approach to challenge this hypothesis would be to compare the expression and function of *Ubx* in association with the number of maxillipeds in different taxonomic groups of crustaceans. The expression pattern of *Ubx* might indeed correlate with morphological pattern of maxillipeds in crustaceans. Another gene related to appendage development in invertebrates and vertebrates is the *Distal-less* (*Dll*) gene. Kato *et al.* [32] established a technique to inject dsRNA into ovulated eggs of the cladoceran crustacean *D. magna*. Injection of *Dll*-specific dsRNA led to *Dll* mRNA degradation. Two dsRNA constructs targeting different regions of the DII gene were administered. Although the survival rates observed were different between the two constructs, the same phenotype of truncated second antenna was observed using these two constructs, suggesting the specific effect of *Dll* RNAi in *D. magna*. In this study, phenotypes of *Dll* RNAi were found to be dose dependent, suggesting that necessary amounts of Dll vary depending on the appendages, organs, and substructure-forming regions.

Methyl farnesoate (MF), the crustacean homolog of the insect juvenile hormone, regulates metamorphosis and is also implicated in the regulation of growth and reproduction in crustaceans [33,34]. Farnesoic acid *O*-methyltransferase (FAMeT) is a key enzyme in MF formation, converting farnesoic acid (FA) to MF. To study the function of this gene in *L. vannamei* (*LvFAMeT*) during molting, Hui *et al.* [35] knocked down the expression of *LvFAMeT* in shrimp using dsRNA injections, preventing treated individuals from advancing to the final stage of the molt cycle and disrupting the expression of the molt-related genes encoding *cathepsin-*L and *hemocyanin*. Subsequently, 100% mortality of the treated individuals was observed (same as in silencing of the ecdysteroid receptor (EcR) primary target *FcE75*), with no death in the control group that was injected with an exogenous non-targeting dsRNA. These results demonstrate that conversion of FA to MF has a crucial role in growth and regulation of molting.

Hypoxia inducible factor 1 (HIF-1) is a transcription factor, which regulates many molecular and physiological responses to hypoxia [36]. HIF-1 is a heterodimer composed of α and β subunits. In a study by Soñanez-Organis *et al.* [37], dsRNA injections targeting *HIF-1* (α or β subunit separately) into *L. vannamei* effectively reduced the transcript levels in gills but not in muscle of shrimp maintained under normoxia and hypoxia. Silencing of either α or β subunits affected the concentrations of glucose and lactate (the substrate and end-product of anaerobic glycolysis respectively) in the hemolymph and gills of shrimps exposed to hypoxic conditions (compared to glucose and lactate levels in the control group), suggesting that a metabolic shift is induced by HIF-1 in response to hypoxia.

Myostatin (MSTN) and GDF11 are closely related members of the transforming growth factor—β (TGF-β) superfamily. This gene was identified in *P. monodon* and its specific function in the regulation of growth was studied through RNAi [38]. In this study *Luciferase-dsRNA* (*Luc-dsRNA*) was used as an exogenous down-regulation control and *β-actin*-dsRNA was used as an experimental technique control. The *pmMstn/Gdf11* gene was down-regulated by tail-muscle injection of sequence-specific dsRNAs. Approximately 40% down-regulation of endogenous *pmMstn/Gdf11* expression was achieved. Positive control animals injected with *β-actin*-dsRNA died within 2–4 days post-injection, indicating that endogenous gene silencing using tail-muscle injection of dsRNA was successful. Shrimp injected with *pmMstn/Gdf11*-dsRNA exhibited a slower growth rate compared with *Luc-dsRNA*- and saline-injected controls. This response is opposite to that seen in higher vertebrates, suggesting that this gene is a positive growth regulator in *P. monodon* [38].

Sarcoplasmic calcium binding protein (SCP) is an invertebrate calcium buffering protein that interacts with two to three calcium ions [39]. White *et al.* identified three SCP variants in the freshwater crayfish *Procambarus* *clarkii* (termed pcSCP1a-c) [40]. Knockdown of *pcSCP1* by dsRNA injections, decreased *SCP* expression by about 50%. The *pcSCP1a* dsRNA that was injected affected the expression of all three *pcSCP1* variants due to sequence similarity to all three, on either side of the variable region. Negative controls were injected with saline or with a 1.85 kb dsRNA from *Xenopus* elongation factor 1 ∝ gene. Crayfish injected with *SCP*-dsRNA were substantially less active than control animals due to a reduction in their muscular activity.

### 2.3. Genes Encoding Eyestalk Neuropeptides

The decapod eyestalk Crustacean Hyperglycemic Hormone (CHH)/Molt Inhibiting Hormone (MIH)/Gonad inhibiting Hormone (GIH) family of neuropeptides is known to regulate important processes such as glucose metabolism, molting and reproduction. CHH neuropeptides were initially detected in the eyestalk X-organ sinus gland neuroendocrine complex (XOSG) [41,42]. Their function was demonstrated in several studies using gene silencing approaches. The first study to report silencing of a decapod-derived neuropeptide was published by Lugo *et al.*, applying RNAi in the shrimp *Litopenaeus schmitti* [43], investigating whether RNAi can inhibit CHH function. Injection of *CHH* dsRNA into the abdominal hemolymph sinuses resulted in undetectable *CHH* mRNA levels within 24 h and a corresponding decrease in hemolymph glucose levels, suggesting that functional gene silencing had occurred. These findings were followed by Tiu and Chan [44] who demonstrated the gonad stimulatory function of the molt-inhibiting hormone (MeMIH-B) in *Metapenaeus ensis* by applying RNAi. The gonad stimulatory function of MeMIH-B was demonstrated by a decline of *vitellogenin* (*Vg*, the precursor of the major yolk protein Vitellin) gene expression in the hepatopancreas and ovary in the MeMIH-B silenced shrimps, reflecting a wider phenomenon of multifunctionality in the CHH superfamily [45].

The role of MIH in regulating molting in the *C. quadricarinatus* was recently demonstrated by RNAi. *In vivo* injections of *C. quadricarinatus MIH* dsRNA resulted in, additionally to eyestalk ablation, acceleration of molt cycles. This acceleration was reflected by a significant reduction (up to 32%) in molt intervals and an increase in molt mineralization index (MMI), which correlated with the induction of ecdysteroid hormones [46].

Tiu *et al.* [47] also identified a CHH-like gene in the white shrimp *L.* *vannamei*, which functions as an ion transport peptide (thus termed *LvCHH-ITP*). Silencing of *LvCHH-ITP* provided molecular evidence to support its osmo-regulatory function. Shrimp injected with high concentration of dsRNA targeting *LvCHH-ITP* died within 24 h. However, no control dsRNA was used to determine whether the death of the animals was caused by *LvCHH-ITP* down-regulation or due to a nonspecific toxic effect of the dsRNA.

A study by Treerattrakool *et al.* [48] provided further support towards the function of GIH in the tiger shrimp *P. monodon*. These authors showed that injections of *P. monodon* *GIH* dsRNA, decrease its transcript levels both in eyestalk ganglia and abdominal nerve cord explant culture and in female *P. monodon*. *GIH* transcript decrease was followed by a precipitous increase in *Vg* transcript level in the ovary of *GIH*-knocked down shrimp individuals, lending support towards the suggested role of GIH as a gonad-inhibiting hormone. In a following study Treerattrakool *et al.* [49] suggested that *GIH*-dsRNA could be used as an alternative method to induce ovarian maturation in female *P. monodon* broodstock, instead of the accepted method of unilateral eyestalk ablation. In a recent study Treerattrakool *et al.* [50] further explored an oral delivery method for GIH silencing (see Section 3.3 and Table 2). However, expression levels of *GIH* in the shrimp fed with *GIH* dsRNA (72%) in comparison with dsRNA injection (32%) showed that oral administration was significantly less effective. Sathapondecha *et al.* [51] studied the effect of *ds-GIH* on the expression of other genes in *P. monodon*. Among the genes that were found to be up regulated in the *ds-GIH* injected females were *thioredoxin* and *transglutaminase*. The expression of the both latter genes was strongly up-regulated in the brains of early-vitellogenic *ds-GIH* treated female shrimp, suggesting that thioredoxin and transglutaminase are both required for an initial stage of vitellogenesis [51].

### 2.4. Receptor-Encoding Genes

Another group of genes studied through silencing in crustaceans encode for receptors, including the growth-related epidermal growth factor receptor *EGFR* [52], the reproduction-related *Vg* receptor (*VgR*, [53]) and the molt-related ecdysteroid receptors (*EcR/RXR*, [54,55]). The role of EGFR in the giant freshwater prawn *Macrobrachium rosenbergii* was revealed by temporarily silencing the transcript through weekly injections of double-stranded *EGFR* RNA. Such treatment resulted in a significant reduction in growth and a delay in the appearance of a male secondary sexual characteristic, the *appendix masculina* [52]. EGFR was also found to play a role in eye development. Similar to the effect of EGFR down regulation in insects [56], *EGFR*-silenced prawns developed abnormal eyes that presented irregular organization of the ommatidia, reflected by unorganized receptor cells occupying large areas of the dioptric portion and by a shortened crystalline tract layer [52]. *VgR* expression was knocked down in the shrimp *P. monodon*, leading to a decrease in VgR protein content in the ovary, and an increase in the hemolymph level of *Vg*. These results support the hypothesis that in shrimp, *Vg* is produced by the hepatopancreas, and is secreted into the hemolymph where it is sequestered into the developing oocytes by *VgR* through receptor-mediated endocytosis [53].

Retinoid X receptor (RXR) is the heterodimeric partner of ecdysteroid receptor and is required for the molting process of arthropods. In Priya *et al.* [55] *RXR* dsRNA was injected into juvenile individuals of the Chinese shrimp *Fenneropenaeus chinensis* resulting with variation in expression levels of two chitinase genes and of the ecdysone inducible gene *FcE75*. In arthropods, chitinases degrade chitin to enable partial breakdown of the exoskeleton prior to molting. The products of hydrolysis are ultimately recycled for the synthesis of a new cuticle [57]. Another important ecdysone inducible gene is the EcR/RXR target gene *E75*, which is one of the primary targets of the ecdysone receptor. A significant reduction in the expression levels of the two chitinase genes and of *FcE75* in RXR-silenced shrimp was detected, suggesting involvement of RXR in the downstream regulation of the ecdysone signaling pathway in decapod crustaceans [55]. In a later research, Priya *et al.* [58] studied the role of FcE75 using the same RNAi technique. *FcE75* dsRNA injections efficiently decreased *FcE75* transcript levels in juvenile *F. chinensis* in a dose dependent manner and completely arrested molting, eventually leading to mortality. Setogenic analysis of the uropods from molt-arrested shrimp showed defective epidermal retraction and poor development of setae and new cuticle.

Recently, Das and Durica [54] reported that RNAi can be successfully applied in the fiddler crab *Uca pugilator* to investigate the morphological and physiological consequences of EcR and RXR silencing during limb regeneration. Disrupting *EcR/RXR* mRNA levels resulted in developmental arrest of growth during early blastemal development and changes in the morphology of limb regeneration. Das and Durica [54] also suggested that RNAi resulted in a systemic effect because contralateral uninjected limbs in experimental animals also exhibited blocked blastemal differentiation. Further strengthening for this notion of a systemic effect came from a significant decrease detected in the ecdysteroid titers in the dsEcR/dsRXR treated crabs and their failure to molt.

In addition to molting, RXR was found to play a role in the reproduction process [59]. Nagaraju *et al.* studied the role of crab RXR in *Carcinus maenas* females via silencing of this gene, using ds-*GFP* as a negative dsRNA control. In this study, the effect of MF was investigated as well. MF was found tostimulate *RXR* and *Vg* expression levels *in vitro* and *in vivo.* Knockdown of *RXR* transcript levels significantly decreased mRNA levels for *RXR* and for *Vg* as well as MF levels in hemolymph and OI (percentage of ovarian weight of the total body weight) of green crab. These findings suggest that RXR may be involved in the MF signaling pathway, either by activating EcR under MF regulation, or by direct interaction with MF [59].

### 2.5. Genes Related to Sexual Differentiation

*Daphnia magna* parthenogenetically produces males in response to environmental signals, rather than the typical genetic sex-specific differentiation common in crustaceans [60]. Still, the conserved gene *Doublesex* (*Dsx*), which has a crucial role in controlling sexual dimorphism, was found in *D. magna*. Two *Dsx* genes have been identified, termed *Dsx1* and *Dsx2*. Silencing of *Dsx1* through dsRNA injections into male embryos resulted in the development of female secondary characteristics and the development of ovaries, while silencing of *Dsx2* did not induce the formation of female characteristics. In insects, there are male-specific and female-specific *Dsx* splice variants, whereas both *Dsx1* and *Dsx2* share the same sequence in *D. magna* males and females. The differences might be attributed to expression levels since *Dsx1* is more strongly expressed in males, which could explain the stronger effect of silencing *Dsx1* compared with *Dsx2* [60]. In malacostracan crustaceans, male sexual differentiation is controlled by the male-specific androgenic gland (AG) [61]. An AG specific insulin-like gene identified in the red-claw crayfish *C.* *quadricarinatus* (*Cq-IAG*), was demonstrated via its silencing to have a vast array of effects on sexual differentiation related characteristics [62]. In *C. quadricarinatus*, sexual plasticity is exhibited by intersex individuals in the form of an active male reproductive system and male secondary sex characters, along with a constantly arrested ovary. Silencing of *Cq-IAG* in intersex individuals induced dramatic sex-related alterations, including male feature feminization, a reduction in sperm production, extensive testicular degeneration, expression of the *Vg* gene, and accumulation of yolk proteins in the developing oocytes. Upon silencing of the gene, AG cells hypertrophied, possibly to compensate for low hormone levels, as reflected by the poor production of the insulin-like hormone (revealed by immunohistochemistry). These results demonstrate both the functionality of *Cq-IAG* as an androgenic hormone-encoding gene and the dependence of male gonad viability on the *Cq-IAG* product.

In *M. rosenbergii* the AG specific insulin-like hormone (Mr-IAG) was shown to be critical for spermatogenesis and the appearance of external sexual characteristics, through silencing of its transcript in juvenile males [63]. Like in *C. quadricarinatus*, silencing of *Mr-IAG* led to considerable hypertrophy of the AG, suggesting a similar mode of feedback regulation in these two decapod species. Later it was shown that *Mr-IAG* silencing in early developmental stages can lead to full-functional sex reversal of males into neofemales [64], leading to a pioneering case of RNAi application in aquaculture (see Section 4).

### 2.6. Genes Related to RNAi Machinery

Among the RNAi components that were identified and characterized in crustaceans are Dicer, Argonaute, transactivating response RNA-binding protein (TRBP) and some other RNAi-related components [65,66,67,68,69,70]. To demonstrate the function of a gene related to the RNAi process, it is possible to silence the silencing machinery itself by injection of long dsRNA for a given target gene (a process also known as “RNAi of RNAi”), as was done for *Argonaute* in *P. monodon* [66] and for *TRBP* (trans-activation response RNA-binding protein) and *eIF6* (eukaryotic initiation factor 6) in *Marsupenaeus japonicas* [69]. 

Argonaute (Pem-ago), a protein constituent of the RNAi machinery was studied in *P. monodon* by Dechklar *et al.* [66]. *P. monodon*’s Argonaute, contains the signature domains PAZ and PIWI which characterize the catalytic components of the RNA-induced silencing complex. *Pem-ago* expression was suppressed by dsRNA administered to shrimp primary cell culture. The *Pem-ago* silenced cells exhibited impaired RNAi function, manifested by rescue of an endogenous gene expression from dsRNA-mediated silencing.

Mj-*TRBP* and Mj*-eIF6*, both are components of RISC [69]. To investigate the role of Mj-*TRBP* and Mj-*eIF6* in the RNAi pathway, shrimps were injected with dsRNA against both genes and *GFP* as control. The silencing of these genes was confirmed by real time RT-PCR and the shrimp were infected with WSSV. Results showed that the DNA copies of WSSV in both the *TRBP*-silenced group and the *eIF6*-silenced group were greatly increased compared to the group injected with *GFP* dsRNA, suggesting their importance in antiviral silencing of shrimp [69]

An array of genes and molecular mechanisms have been investigated within a short time frame in crustaceans, using RNAi techniques to facilitate gene-specific knockdown (Table 1). Most studies were performed in species of the commercially important order *Decapoda*. The ability to further accelerate the pace at which genes are being investigated in crustaceans using RNAi approach necessitates a more efficient administration method, more robust than injections, such as used in *C. elegans*, through feeding [22].

## 3. Study of the Crustacean Innate Immune System Using RNAi

RNAi is hypothesized to have evolved, in part, as an anti-viral mechanism [71]. Hence, harnessing RNAi to combat viruses is a reasonable choice of weaponry. Due to the immense importance of crustaceans in aquaculture and the fact that the crustacean aquaculture industry is struggling with severe viral outbreaks [72], application of RNAi for combating viruses is a field of study practiced by many research groups. In fact—half of the studies published thus far, which involve RNAi in crustaceans (see Section 3.1 and Table 3), including most of the studies which explored delivery methods of silencing agents into crustaceans (see Section 3.2 and Table 2)—investigated the option of using RNAi either to combat viruses or to study the mechanism underlying the innate immune system in crustaceans. Recent reviews on this field of research were published by Hirono *et al.* [73] and La Fauce and Owens [74], and prominent studies conducted in this field are summarized in Table 2 and Table 3. This section will provide key examples of using RNAi to study and mitigate viral infections in crustaceans. Also summarized in this section are studies that employed alternative administration routes to dsRNA injections (Section 3.2 and Table 2), since these were performed in the context of immune system studies.

### 3.1. Crustacean Innate Immune System Study through RNAi

Dicer is an endoribonuclease which serves as a key component of the RNAi mechanism [75]. Dicer-1 silencing in the tiger shrimp *P. monodon* increased susceptibility to gill-associated viral infections [71], suggesting that like in other organisms RNAi is crucial for anti-viral innate immunity in crustaceans. In a study by De la Vega *et al.* [76], two antimicrobial peptides were silenced in the white shrimp *L. vannamei*, followed by challenging with pathogenic strains of bacteria, fungi and viruses. While mortality rates soared when treated shrimps were challenged with bacteria and fungi, this was not the case when challenged with white spot syndrome virus (WSSV). The researchers concluded that silencing activated the sequence-independent innate anti-viral immune response, leading to increased protection from WSSV infection. Similarly, non-specific engagement of the silencing mechanism through injection of dsRNA of various sizes ranging between 50 bp and 200 bp, reduced mortality rates of WSSV challenged *L. vannamei* individuals, while siRNA (20–25 bp) injection did not reduce mortality rates [67].

**Table 2:** Summary of RNAi administration methods in crustaceans.

Delivery method	Model Organinsm	Order	Year	Authors	Title
Injection, electroporation and transfection of DNA with promoter and antisense	*Litopenaeus vannamei*	*Decapoda*	2005	Sun *et al.*	Evaluation of methods for DNA delivery into shrimp zygotes of *Penaeus* (*Litopenaeus*) *vannamei*
Transfection of DNA with promoter and antisense	*Litopenaeus vannamei*	*Decapoda*	2005	Lu and Sun	Viral resistance in shrimp that express an antisense Taura syndrome virus coat protein gene
Chitosan nanoparticles	*Macrobrachium rosenbergii*	*Decapoda*	2008	Anas *et al.*	Chitosan as a wall material for a microencapsulated delivery system for *Macrobrachium rosenbergii* (de Man) larvae
Oral delivery of dsRNA expressing bacteria	*Penaeus monodon*	*Decapoda*	2008	Sarathi *et al.*	Oral Administration of Bacterially Expressed VP28dsRNA to Protect *Penaeus monodon* from White Spot Syndrome Virus
Oral delivery of dsRNA expressing bacteria enriched *Artemia*	*Penaeus monodon*	*Decapoda*	2013	Treerattrakool *et al.*	Silencing of gonad-inhibiting hormone gene expression in *Penaeus monodon* by feeding with GIH dsRNA enriched *Artemia*

**Table 3:** Study of the crustacean innate immune system using RNAi.

Field of Research	RNAi administration method	Model Organinsm	Order	Year		Authors	Title
Anti bacterial	dsRNA injection	*Pacifastacus leniusculus*	*Decapoda*	2007		Liu *et al.*	Phenoloxidase is an important component of the defense against *Aeromonas hydrophila* infection in a crustacean, *Pacifastacus leniusculus*
Anti bacterial	siRNA injection	*Marsupenaeus japonicus*	*Decapoda*	2008		Zong *et al.*	Regulation of phagocytosis against bacterium by RabGTPase in shrimp *Marsupenaeus japonicus*
Anti bacterial and anti viral	dsRNA injection	*Litopenaeus vannamei*	*Decapoda*	2008		De la Vega *et al.*	Anti-lipopolysaccharide factor in *Litopenaeus vannamei* (LvALF): A broad spectrum antimicrobial peptide essential for shrimp immunity against bacterial and fungal infection
Anti bacterial, antiviral and coagulation	dsRNA injection	*Marsupeneus japonicus*	*Decapoda*	2008		Maningas *et al.*	Essential function of transglutaminase and clotting protein in shrimp immunity
Anti bacterial	dsRNA injection	*Marsupeneus japonicus*	*Decapoda*	2009		Fagutao *et al.*	Increased bacterial load in shrimp hemolymph in the absence of prophenoloxidase
Anti bacterial	dsRNA injection	*Penaeus monodon*	*Decapoda*	2009		Amparyup *et al.*	Two prophenoloxidases are important for the survival of *Vibrio harveyi* challenged shrimp *Penaeus monodon*
Anti bacterial and anti fungal	dsRNA injection	*Litopenaeus vannamei*	*Decapoda*	2009		Shockey *et al.*	The role of crustins in *Litopenaeus vannamei* in response to infection with shrimp pathogens: An *in vivo* approach
Anti viral	siRNA injection	*Penaeus monodon*	*Decapoda*	2005		Westenberg *et al.*	siRNA injection induces sequence-independent protection in *Penaeus monodon* against white spot syndrome virus
Anti viral	Injection, electroporation and transfection of DNA with promoter and antisense	*Litopenaeus vannamei*	*Decapoda*		2005	Sun *et al.*	Evaluation of methods for DNA delivery into shrimp zygotes of *Penaeus* (*Litopenaeus*) *vannamei*
Anti viral	Transfection of DNA with promoter and antisense	*Litopenaeus vannamei*	*Decapoda*		2005	Lu and Sun	Viral resistance in shrimp that express an antisense Taura syndrome virus coat protein gene
Anti viral	dsRNA injection	*Penaeus monodon*	*Decapoda*		2006	Assavalapsakul* et al.*	Identification and characterization of a *Penaeus monodon* lymphoid cell-expressed receptor for the yellow head virus
Anti viral	siRNA injection	*Marsupenaeus japonicus*	*Decapoda*		2007	Xu *et al.*	Silencing shrimp white spot syndrome virus (WSSV) genes by siRNA
Anti viral	dsRNA injection	*Marsupenaeus japonicus*	*Decapoda*		2007	Li *et al.*	β-integrin mediates WSSV infection
Anti viral	siRNA injection	*Marsupenaeus japonicus*	*Decapoda*		2007	Wu *et al.*	Antiviral phagocytosis is regulated by a novel Rab-dependent complex in shrimp *Penaeus japonicus*
Anti viral	siRNA injection	*Marsupenaeus japonicus*	*Decapoda*		2008	Xu *et al.*	Novel function of QM protein of shrimp (*Penaeus japonicus*) in regulation of phenol oxidase activity by interaction with hemocyanin
Anti viral	siRNA injection	*Marsupenaeus japonicus*	*Decapoda*		2008	Wang *et al.*	Requirement for shrimp caspase in apoptosis against virus infection
Anti viral	dsRNA injection	*Penaeus monodon*	*Decapoda*		2008	Ongvarrasopone *et al.*	Suppression of PmRab7 by dsRNA inhibits WSSV or YHV infection in shrimp
Anti viral	dsRNA injection	*Penaeus monodon*	*Decapoda*		2008	Su *et al.*	A key gene of the RNA interference pathway in the black tiger shrimp, *Penaeus monodon*: Identification and functional characterisation of Dicer-1
Anti viral	dsRNA injection	*Litopenaeus vannamei*	*Decapoda*		2008	Rijiravanich *et al.*	Knocking down caspase-3 by RNAi reduces mortality in Pacific white shrimp *Penaeus* (*Litopenaeus*) *vannamei* challenged with a low dose of white-spot syndrome virus
Anti viral	Oral delivery of dsRNA expressing bacteria	*Penaeus monodon*	*Decapoda*		2008	Sarathi *et al.*	Oral administration of bacterially expressed VP28 dsRNA to protect *Penaeus monodon* from White Spot Syndrome Virus
Anti viral	siRNA and dsRNA injection	*Litopenaeus vannamei*	*Decapoda*		2010	Labreuche *et al.*	Non-specific activation of antiviral immunity and induction of RNA interference may engage the same pathway in the Pacific white leg shrimp *Litopenaeus vannamei*
Anti viral	dsRNA injection	*Penaeus monodon*	*Decapoda*		2011	Woramongkolchai *et al.*	The possible role of penaeidin5 from the black tiger shrimp, *Penaeus monodon*, in protection against viral infection
Anti viral	Review on the study of innate immune system in crustaceans using RNAi				2011	Hirono *et al.*	Uncovering the mechanisms of shrimp innate immune response by RNA interference
Anti viral	Review on RNAi in crustaceans with emphasis of antiviral capabilities				2012	La Fauce and Owens	RNA interference with special reference to combating viruses of crustacea
Anti viral	dsRNA injection *Litopenaeus vannamei Decapoda*				2013	Lin *et al.*	Characterization of white shrimp *Litopenaeus vannamei* integrin b and its role in immunomodulation by dsRNA-mediated gene silencing

In another study in *L. vannamei*, silencing crustin, an anti-microbial peptide suspected to also inhibit fungal growth—was followed by injection of pathogenic bacteria or fungi. While mortality rates rose in crustin-depleted individuals challenged with bacteria, no change was observed in crustin-depleted individuals challenged with fungi [77]. The sequence-independent immune response was not tested in this study. In yet another study, the transcript level of penaeidin5, an antimicrobial peptide of the marine shrimp *F. chinensis*, was elevated 24 h after WSSV challenging, followed by a sharp decline after 48 h. Since transcript levels were correlated with viral titer, the researchers sought to investigate the anti-viral function of penaeidin5. Silencing penaeidin5 increased the transcript level of VP28, a coat protein of WSSV [78]. This study provided the first evidence for a penaeidin that is anti-viral. The clotting mechanism, the first line of defense in crustaceans [79], was also implicated in anti-viral defense. Silencing of two components of the clotting mechanism followed by challenging the treated shrimp with bacteria and viruses, both resulted in higher mortality rates in the silenced individuals [80].

Caspase-3 is a cysteine protease which is the effector in apoptosis, necrosis and inflammation [81]. Silencing of caspase-3 in the marine shrimp *Marsupenaeus japonicus* through siRNA injections inhibited WSSV-induced apoptosis and led to an increase in observed viral copy numbers, suggesting that apoptosis is an anti-viral mechanism in this species [82]. This notion was strengthened by silencing of caspase-3 in the white shrimp *L. vannamei* (through dsRNA injections) which resulted in increased mortality following challenging with WSSV [83]. These results are contradictory to the same intervention in mammalian cell lines, where caspase-3 knock-down by siRNA impaired influenza propagation [84]. This contradiction emphasizes the differences in anti-viral defense mechanisms between arthropods and vertebrates.

Anti-bacterial as well as anti-viral phagocytosis, which precedes caspase-3 activity, was studied in the marine shrimp *M. japonicus*. Silencing a Rab GTPase—a major component of endocytosis as well as phagocytosis—decreased phagocytosis activity in *vibrio* sp. challenged individuals [85] and increased the copy number of WSSV with which treated shrimp were challenged [86], suggesting that a Rab-dependent complex plays a role in anti-bacterial as well as anti-viral phagocytic activity in crustaceans. Ongvarrasopone *et al.* [87] found that silencing Rab GTPase in *P. monodon* reduces proliferation of both WSSV- and yellow head virus (YHV) in challenged individuals, suggesting that tackling the phagocytosis process might be beneficial against an array of viruses. Through incubating YHV with membrane extracts of *P. monodon* lymphoid organ, a 65 kDa receptor was identified. Silencing of the receptor followed by challenging with YHV completely inhibited the entry of the virus, suggesting that this is the receptor employed by the virus to enter the host cells [88].

Prophenol oxidase (proPO) is the terminal enzyme in the melanization cascade, an important innate immune system in crustaceans [89]. ProPO silencing in the shrimp *M. japonicus* induced mortality due to bacterial build up, which was counteracted by antibiotic treatment. A microarray revealed that expression levels of several antimicrobial peptides were reduced in proPO silenced individuals [90]. Two proPO genes were identified in the closely related tiger shrimp *P. monodon*. Silencing of each of the two, followed by challenging with the pathogenic bacterium *Vibrio harveyi* increased mortality, suggesting that both genes are active components of the melanization process in *P. monodon* [91]. Silencing of proPO in the crayfish *Pacifastacus leniusculus* followed by challenging with a pathogenic bacterium increased mortality, while silencing of pacifastin, an inhibitor of the crayfish proPO activation cascade, delayed mortality [92]. Silencing of the QM protein, a tumor suppressor candidate showed that it regulates proPO levels in the shrimp *M. japonicas* [93].

Through co-immunoprecipitation, *β*-integrin was found to interact with a WSSV coat protein in *M.* *japonicus*. Silencing *β*-integrin reduced mortality in WSSV-challenged individuals [94], suggesting *β*-integrin is yet another key for viral invasion into host cells. An array of immuno related genes, exhibited varying expression levels following *β*-integrin silencing in *L. vannamei*. *β*-integrin involvement was suggested in: proPO activation, phagocytosis, and the antioxidant system for immunomodulation in shrimp [84].

In summary, within a short period of time, RNAi has become a key method to explore an array of cellular processes involved in anti-viral defense mechanisms in non-model crustaceans of commercial importance. This exemplifies the RNAi ease-of-use and the vast interest in this method for paving new application avenues in this field of research.

### 3.2. Anti Viral-Related Administration Methods

Administration methods in crustaceans have mainly been explored in the context of viral defense. Attempts were made to vaccinate crustaceans by administering DNA constructs, which express viral capsid genes [95,96], as well as silencing compounds (namely dsRNA, siRNA and shRNA). Sun *et al.* [8] explored an array of methods for transfecting white shrimp (*L. vannamei*) zygotes with DNA constructs of a taura syndrome virus (TSV) coat protein anti-sense, transcribed under the control of beta-actin promoter. The methods included microinjection, electroporation and transfecting with a commercially available transfecting reagent. The transfecting reagent proved to be the most efficient method as it is up-scalable and simple and yields high hatching rate, gene transfer efficiency, and survival rate [7]. The major drawback of this method is that unlike temporal silencing compounds (dsRNA/siRNA/shRNA), DNA transfection might produce a genetically modified organism (GMO).

Sarathi *et al.* [97] explored oral administration methods including dsRNA-producing bacteria and dsRNA-chitosan nanoparticles. The dsRNA targeted the coat protein of the WSSV and the bioassay included mortality rates following oral challenging with WSSV. Mortality rates were reduced by 37% and 68% in the dsRNA-producing bacteria and dsRNA-chitosan nanoparticles—fed individuals, respectively. Anas *et al.* [98] further characterized the properties of chitosan nanoparticles and concluded that they are suitable for encapsulating drugs for oral delivery into *M. rosenbergii* larvae. While this approach proved useful to some extent, only one very recent report has examined the option of using oral delivery to silence a gene systemically in crustaceans (summarized in Section 3.3). This method might only be useful for protecting against water-borne viruses, rather than for silencing of endogenous genes. Contradictory to the findings by Sarathi *et al.* [97], when *P. monodon* individuals were challenged with a lethal dose of a gill associated virus, mortality was not reduced when fed with dsRNA-producing bacteria, while intramuscular dsRNA injections did lead to a decrease in mortality [99].

Using the same WSSV coat protein as the silencing target, Xu *et al*. [100] showed that a 21 nucleotide-long siRNA is capable of eradicating WSSV from infected *Penaeus* *japonicus* shrimp following three injections. These researchers showed that a single mutation eliminates the siRNA activity. Contrary to the above, Westenberg *et al.* [101] found that in *P. monodon* siRNA acts in a non-specific manner, reducing mortality of WSSV-challenged shrimp individuals, regardless of whether the siRNA is designed to silence expression of WSSV coat proteins.

In summary, the predominance of viral-related studies that employ RNAi in crustaceans reflects the need of the aquaculture industry to mitigate the ever-increasing threat of viral-induced collapses of cultured crustacean ponds. The study of administration methods in this context might pave the way towards a more robust use of RNAi in various fields of study in crustaceans.

### 3.3. Oral Administered Silencing of a Systemic Gene

In a recent study, an attempt was made to silence an endogenous gene in crustaceans through oral delivery [50]. In this study previously successful silencing through dsRNA injections of GIH in *P. monodon* [48] was repeated, this time by feeding with *Artemia salina* enriched with dsRNA expressing bacteria. Although a significant decrease in *GIH* transcript levels was observed, it was much less pronounced than with the dsRNA injections. This study indicates that it is feasible to induce silencing in crustaceans through orally-administered silencing compounds although further study is required to increase the efficiency. One plausible route would be the use of siRNA instead of dsRNA.

## 4. The First Case of a Biotechnological Use of Gene Silencing in the Aquaculture Industry (Crustacean Monosex Culture)

The opportunities opened by gene silencing in crustaceans are immense and indeed the first commercialized RNAi based biotechnology implemented in the aquaculture industry was achieved in a prawn (decapod crustacean) culture as described below.

### 4.1. Sexual Dimorphism and Monosex Culture of Prawns

Gender, sexual maturity and reproductive activity are among the factors affecting growth rates of crustaceans under aquaculture conditions [102,103,104]. This is most notable in crustacean species which exhibit bimodal growth patterns in which males grow faster than females or vice versa [102], among these are the most popular decapod cultured groups (shrimp, prawns, crayfish and crabs). The bimodal growth phenomenon has led to the assumption that culturing each gender separately, as commonly done in agriculture in the major cases of animal husbandry (e.g., cattle and poultry) might be of advantage for crustacean cultures as well. This assumption is based on the understanding that differences between males and females in terms of growth rate, alimentary needs and behavioral patterns dictate the need to establish management systems specifically tailored to one sex or the other. Moreover, non-breeding monosex populations may divert energy from reproduction to growth. Based on these assumptions, attempts have been made to apply the monosex husbandry into crustacean aquaculture [105,106].

The giant freshwater prawn, *M. rosenbergii* (de Man), which is a popular aquaculture species, displays clear dimorphic growth in which males reach significantly larger sizes than females [107]. Thus, a preliminary attempt to grow the prawns under gender separation was made in a small-scale cage-culture system, clearly demonstrating the advantage of all-male yields compared to a mixed population [108]. In addition to the higher yields, the all-male culture of *M. rosenbergii* proved later to be economically beneficial with a 60% income increase under Indian conditions [109]. The unmet need thus was a viable biotechnology for producing monosex prawn populations, and this was established by manipulating the endocrinic control of sexual differentiation in crustaceans in the androgenic gland [110].

### 4.2. The Androgenic Gland and Its Secretion

The androgenic gland (AG) was suggested by Charniaux-Cotton [111] to mediate sexual differentiation in crustaceans and nowadays it is accepted that the gland plays a central role in this function [112,113,114]. Touir [115] described the effects of the AG on both primary and secondary male characteristics in a number of decapod crustaceans, and Taketomi *et al.* [116] showed the same using AG extracts in the crayfish *Procambarus clarkii*. More recently, it was shown that AG implantation into females of the crayfish *C. quadricarinatus* and the mud crab *Scylla paramamosain* inhibited vitellogenesis, resulted in ovarian regression with degeneration oocytes and promoted growth [117,118].

The ultrastructure of a decapod crustacean AG cell resembles that of a vertebrate protein-producing cell rather than that of a steroid-producing cell, thus it appears that unlike in vertebrates, it is proteins rather than steroids that are responsible for the control of sexual differentiation [119]. Histological evidence [120] and changes in total protein content in specific AG cell types [121] in *M. rosenbergii* support this notion. This is further supported by purification, identification and full DNA sequencing of the AG hormone in isopods [122,123]. Similarly, in recent years, insulin-like AG hormone encoding transcripts were discovered and fully sequenced in all economically important groups of decapod crustaceans including shrimp, crabs, prawns and crayfish [63,64,124,125,126].

### 4.3. RNAi Based Biotechnology for All-Male Culture

Following the discovery of the insulin-like AG hormone encoding transcript in the prawn *M. rosenbergii* (*Mr-IAG*), gene silencing through RNAi was used to knock down this crustacean sexual differentiation-related gene [63]. When performed at an early developmental stage of juvenile males, identified through the use of molecular sex markers [127], *Mr-IAG* silencing induced a full and functional sex reversal of males into neo-females. Successfully mated with untreated males, the neo-females produced all-male progeny [16]. This marked the first commercialization of the process and establishment of a technology that does not involve the use of chemicals/hormones and does not involve genetic modification of the target organism. Since the intervention is temporal and performed on parent prawns, it is not transmissible to next generations and thus free of the regulatory hurdles required from genetically-modified crops [128]. Based on these advantages and with further developments of delivery methodologies, in the coming years we will probably see gene silencing applications emerging in many aspects of crustacean culture and aquaculture in general.

## 5. Conclusions

Within the short period of time since its discovery, RNAi has served to enhance basic scientific knowledge through the discovery of the function of a wide array of genes and molecular mechanisms in crustaceans. RNAi has also become a key method to explore cellular processes involved in anti-viral defense mechanisms in non-model crustaceans of commercial importance. Indeed, the first commercialized RNAi-based biotechnology in the aquaculture industry was achieved in prawns (decapod crustacean).

To further accelerate the pace at which RNAi can be used in the investigation of genes, in various fields of study in crustaceans, a more efficient administration method tailored to these organisms is required.

This review exemplifies the ease-of-use of RNAi, the vast interest in this method and the immense opportunities opened by gene silencing in the field of crustacean research and development.
